# A Cross-Sectional Study on the Relationship Between Social Media Use and Frailty Among the Older People in Japan

**DOI:** 10.3390/ijerph22020142

**Published:** 2025-01-22

**Authors:** Yuki Nakada, Yuna Seo

**Affiliations:** Department of Industrial and Engineering Systems, Graduate School of Science and Technology, Tokyo University of Science, Noda 278-8510, Chiba, Japan

**Keywords:** frailty, social media use, older adults, social connectivity

## Abstract

This study investigates the relationship between social media use and frailty in older adults, focusing on the influence of social media engagement and various frailty-related factors. A survey was conducted with 103 participants aged 65 and above, who completed a questionnaire on their social media usage, psychological well-being, dietary habits, physical activity, sleep patterns, and social interactions. Frailty was assessed using the Kihon Checklist, categorizing participants into non-frailty, pre-frailty, and frailty groups. The analysis was conducted using ordinal logistic regression to examine the relationship between social media usage and other frailty-related factors (e.g., psychological factors, sociality, diet, and exercise) with frailty status. The findings revealed that social media engagement was significantly associated with frailty status, with higher levels of engagement linked to reduced frailty. Specifically, participants who reported higher levels of social media interaction also reported better psychological well-being, increased social interaction, and greater engagement in physical and leisure activities. These results suggest that social media use may have a positive impact on frailty, potentially by enhancing social connectivity and promoting healthier lifestyle choices in older adults. Further research is needed to explore the mechanisms through which social media can mitigate frailty and promote healthy aging.

## 1. Introduction

Frailty is a clinical condition characterized by a decline in physical, cognitive, and social functions, placing older individuals at greater risk of adverse health outcomes [1]. As people age, their ability to recover from physical or psychological stress diminishes, leading to a transitional stage between health and disability known as frailty. This condition is significant in the context of aging because it not only increases the likelihood of hospitalization and mortality but also decreases quality of life, making it a critical focus for aging populations worldwide. Frailty can be categorized into three primary types: physical frailty, which involves the weakening of muscular strength and mobility; cognitive frailty, which is linked to declining mental function; and social frailty, marked by reduced social engagement and isolation.

Globally, frailty assessments such as the Fried phenotype and the Frailty Index (FI), which focus on physical aspects, are commonly used [2]. However, research indicates that social participation can play a critical role in preventing functional decline and mitigating health disparities among older adults. In line with these findings, Japan’s approach to frailty countermeasures adopts a primary healthcare perspective, integrating physical, cognitive, and social dimensions to enhance the well-being of its aging population [3].

The prevalence of frailty in Japan, as assessed using the Fried phenotype, is 8.7%, which is relatively lower compared to other countries. Additionally, the prevalence of pre-frailty is 40.8%, while 50.5% of older adults are considered robust [4]. To address frailty, Japan has implemented a national initiative to identify older adults at high risk of requiring long-term care and to connect them with preventive care programs. A key tool in this initiative is the ‘Kihon Checklist,’ developed by the Ministry of Health, Labour and Welfare, which evaluates physical, cognitive, and social functions comprehensively [5]. Satake further adapted this checklist to assess frailty, reflecting a holistic approach that encompasses more than just physical health.

Among these, social frailty is particularly important, as strong social connections are known to play a protective role in maintaining overall health and well-being [6]. Social isolation, a key contributor to social frailty, has been associated with increased rates of physical decline and cognitive impairment [7]. Given the importance of maintaining social relationships to prevent frailty, exploring tools that enhance social interaction among older people is critical.

Social media has become a popular tool for building and maintaining social relationships. In Japan, its use is increasingly widespread among older adults, with 71.7% of those aged 60–69 and 60.7% of those aged 70 and older engaging with platforms such as Facebook and LINE [8]. These platforms are primarily used to interact with family and acquaintances through text messaging, video calls, and group chats. Additionally, older adults often share and view photos, participate in community groups, follow news updates, and engage in hobby-related activities such as gardening, cooking, or travel discussions. For the purposes of this study, ’social media use’ is broadly defined as engaging with these platforms for virtual interaction, content sharing, and community participation. Among older adults, social media can help bridge gaps caused by geographical distances or limited mobility, providing opportunities for connection with friends, family, and communities [9,10]. While its potential to reduce social isolation is well documented, its relationship with frailty remains underexplored.

In this pilot study, we hypothesize that there should be a correlation between frailty and social isolation, and that social media usage could have the potential to alleviate social isolation. Therefore, the purpose of this study was to analyze the relationship between social media usage and frailty among older people.

## 2. Theoretical Background

Frailty, a multidimensional syndrome encompassing physical, psychological, and social health, has emerged as a critical area of research in aging populations. This condition is characterized by an increased vulnerability to adverse health outcomes, including falls, hospitalization, and mortality. The literature highlights frailty as a dynamic process influenced by both intrinsic and extrinsic factors, necessitating a comprehensive approach to understanding its determinants. While existing studies have shed light on various determinants of frailty, gaps remain in understanding the interplay between these factors, particularly the role of social media use. Most research has focused on individual variables in isolation, neglecting the synergistic effects of psychological, social, and lifestyle factors. This study aims to address these gaps by examining the comprehensive influences on frailty, with a particular focus on the underexplored relationship between social media use and frailty in older adults. By integrating these dimensions, this research seeks to contribute to the development of holistic frameworks for frailty prevention and intervention.

### 2.1. Theoretical Models of Frailty

The conceptualization of frailty has evolved significantly over the past few decades. Early models focused predominantly on physical decline, such as Fried’s frailty phenotype, which emphasizes weight loss, exhaustion, weakness, slowness, and low physical activity [11]. However, recent frameworks, including the multidimensional model, recognize the interplay of psychological and social factors alongside physical health [12,13]. This holistic perspective aligns with the growing acknowledgment that frailty cannot be fully understood without considering lifestyle and environmental influences.

### 2.2. Social Media Use and Frailty

Social media use, a relatively novel variable in frailty research, has garnered attention for its dual role in influencing social connectivity and psychological well-being. Studies suggest that active engagement on social media can mitigate feelings of loneliness and promote social interaction, which are protective against frailty [14]. Conversely, excessive use or negative experiences, such as cyberbullying or social comparison, may exacerbate psychological stress, a known contributor to frailty [15]. Despite these findings, the direct relationship between social media use and frailty remains underexplored, with limited empirical evidence addressing how digital interactions influence physical, psychological, and social dimensions of frailty.

### 2.3. Psychological and Social Factors

Psychological well-being, including subjective happiness and resilience, has been shown to buffer against frailty by fostering positive health behaviors and enhancing social engagement [16]. Loneliness, on the other hand, is a significant predictor of frailty, with studies demonstrating its adverse effects on mental and physical health outcomes [17]. Social participation, whether through community activities or leisure pursuits, has been identified as a protective factor, promoting cognitive stimulation and reducing the risk of frailty [18]. These findings underscore the importance of integrating psychological and social variables into frailty research to develop effective interventions.

### 2.4. Lifestyle Factors: Diet, Exercise, and Sleep

Lifestyle factors are well-established determinants of frailty. Adequate nutrition, particularly protein and micronutrient intake, plays a pivotal role in maintaining muscle mass and physical function [19]. Exercise, especially strength and balance training, has been consistently associated with reduced frailty risk by improving physical performance and resilience [20]. Sleep quality, often overlooked, also influences frailty by affecting cognitive function, emotional well-being, and physical health [21]. The interplay of these factors highlights the need for multidimensional assessments to capture the complexity of frailty.

### 2.5. Oral Health and Frailty

Oral health, specifically the number of remaining teeth and regular dental check-ups, has emerged as a novel area of interest in frailty research. Studies have linked poor oral health to increased risks of malnutrition, physical decline, and social isolation, all of which contribute to frailty [22]. Preventive measures, such as regular dental visits, have been associated with better health outcomes and reduced long-term care costs, further emphasizing the importance of oral health in frailty prevention [23].

## 3. Materials and Methods

### 3.1. Study Sample

In this pilot study, we defined older individuals as those aged 65 and over, in line with the definition set by the World Health Organization (WHO). We conducted a pretest via an online survey to evaluate the clarity and comprehensibility of the questionnaire items and to test the variables affecting frailty. Additionally, the pretest aimed to assess the actual usage of social media among older adults to refine the final survey instrument. In the pretest, we distributed a questionnaire to 365 older people aged 65 and over and used the data from 333 participants who agreed to participate in the study for analysis.

The main survey was conducted via mail. We targeted older people individuals residing in Sumida Ward in Tokyo and Matsudo City in Chiba Prefecture, both of which are part of the Tokyo Metropolitan Area, including Saitama Prefecture, Chiba Prefecture, Tokyo, and Kanagawa Prefecture [24]. These areas have large populations, with Matsudo City housing approximately 500,000 residents, 25.8% of whom are elderly (as of 2024) [25], and Sumida Ward with around 200,000 residents, 20.6% of whom are elderly (as of 2024) [26]. These figures show that the elderly population in these areas closely aligns with the national average in Japan. Older adults in Japan’s metropolitan areas exhibit distinct lifestyle characteristics influenced by urban living. According to the Japanese White Paper on Aging society, many seniors live in nuclear households or alone, leading to increased risks of social isolation [13]. While public transportation accessibility supports mobility, urban seniors often face limited opportunities for community engagement. Additionally, dietary habits and meal consistency vary, with some skipping meals due to busy schedules or health conditions, potentially contributing to frailty.

Addresses were collected from each municipality after receiving approval to perform this research. We sent questionnaires to the addresses of 1333 older people and received valid responses from 103 individuals, who provided informed consent.

This study was approved by the Research Ethics Committee of Tokyo University of Science for Medical and Biological Research Involving Human Subjects [#23055], which waived the need for informed consent for ethical approval and consent to participate.

### 3.2. Measures

The data for this study were collected through a mailed survey, where participants were asked to respond to questions related to their use of social media, frailty, and other factors known to influence frailty, including psychological factors, sociality, dietary habits, sleep, and demographic characteristics. These variables were carefully selected to provide a comprehensive view of the influences on frailty in older adults. The questionnaire used for this survey is provided in the Supplementary Materials (Table S1).

Frailty was assessed using a well-established tool, the Kihon Checklist, as described by Satake [12], which is widely used for frailty assessment in older adults in Japan. The checklist includes 25 items that assess physical, psychological, and social health. Based on the total score, participants were classified into three categories: non-frailty (score ≤ 3), pre-frailty (score 4–7), and frailty (score ≥ 8). The validity of this checklist is supported by its strong correlation with adverse health outcomes in older adults [5]. Frailty is recognized as a complex, multidimensional condition that is influenced by both intrinsic factors (e.g., age, comorbidities) and extrinsic factors (e.g., social engagement, lifestyle habits).

Research has shown that social media usage can both mitigate and exacerbate feelings of social isolation, which is a key contributor to frailty. The psychological benefits of social media, such as providing a platform for self-expression and maintaining social connections, have been linked to improved mental health and social well-being, while excessive use may lead to social comparison and anxiety [9,10].

To examine social media usage, questions were included to measure the frequency of social media use, social media engagement, interaction, communication, etc. For example, questions such as “I often like and share on social media” and “I think I can express my feelings and thoughts on social media” assess frequency and interactions on social media platforms. The responses were based on a 4-point Likert scale: 1 (strongly disagree) to 4 (strongly agree). This measure was chosen to capture both the active engagement with social media and the perceived social support it may provide, which has been linked to mental well-being and social connectivity [9].

Dietary habits are widely recognized as key determinants of frailty in older adults. Poor nutrition has been linked to a higher risk of frailty due to its impact on physical function and immune health [27]. Previous studies have shown that reducing the frequency of meals from three per day increases the risk of mortality [28]. To assess meal consistency and the potential risk of undernutrition or inadequate calorie intake—factors that may contribute to frailty—we included the question, “Do you usually not eat three meals a day?” (yes/no). Adequate nutrient intake, particularly of protein and essential micronutrients, has been demonstrated to play a critical role in preventing muscle loss and maintaining physical function in aging populations [29].

Oral health was evaluated using two items. First, participants were asked whether they had 19 or fewer teeth or 20 or more teeth. Research has shown that individuals aged 65 years or older with 19 or fewer teeth are at a higher risk of falls compared to those with 20 or more teeth [30]. Sato further suggested that having 19 or fewer teeth is associated with an increased risk of various health conditions [31]. Second, we included the question, “Do you go for a dental check-up once every six months?” Previous studies have demonstrated that regular dental check-ups within six months are associated with reduced long-term care (LTC) costs [32]. This indicates that regular dental visits may serve as a preventive measure against frailty and the need for care by contributing to overall oral health and reducing LTC costs, which often represent the ultimate outcome of caregiving status.

Physical activity is another major factor influencing frailty, with regular exercise shown to reduce the risk of developing frailty by improving strength, balance, and overall physical function [33]. To measure this, we included the question, “Do you consciously exercise for your health?”, which assesses whether participants engage in regular physical activity, an important factor in maintaining independence and reducing frailty risk. The positive relationship between physical activity and frailty is well documented, with exercise interventions shown to improve frailty outcomes in older adults [33].

Sleep is an essential component of health that impacts multiple aspects of frailty, including physical function, cognitive health, and emotional well-being. Poor sleep quality has been shown to contribute to frailty, particularly in older adults who may experience disrupted sleep patterns due to aging or health conditions [34]. Questions like “Do you get enough rest from sleep over the past month?” and “Are you satisfied with your overall sleep?” are designed to capture both the quantity and quality of sleep, which are important predictors of frailty.

Subjective well-being, including self-reported happiness, is a known correlation of frailty. Research has suggested that positive emotions and a sense of purpose can buffer against frailty by enhancing resilience and encouraging social engagement [35]. The question, “Do you think you are happy?” was included to measure the participants’ overall sense of well-being, which has been linked to better health outcomes in older adults.

Loneliness, particularly when coupled with social isolation, is a major predictor of frailty in older adults. Research indicates that feelings of alienation and loneliness increase the risk of frailty by exacerbating psychological stress and reducing social support, which are key factors in maintaining physical and mental health [36]. In this study, loneliness was measured using questions such as “Do you trust other people?” and “Do you feel isolated from others?” to capture both interpersonal trust and the subjective experience of social isolation.

Participation in social activities has been shown to reduce frailty risk by enhancing social connections and providing opportunities for physical activity and mental stimulation. Engaging in community activities has positive effects on cognitive function and overall well-being, acting as a protective factor against frailty [37]. The question, “Do you participate in community activities a lot?” assesses the extent of social engagement and its potential protective effects on frailty.

Leisure activities, particularly those involving social interaction or physical activity, are also important for preventing frailty. The act of going out for personal hobbies and entertainment can contribute to physical and cognitive well-being, thus preventing social isolation and promoting resilience [38]. Questions like “Do you actively go out for non-work activities?” and “Do you often engage in your favorite leisure activities?” were designed to assess both the frequency and enjoyment of leisure activities, which have been associated with greater happiness and reduced frailty risk in older adults [39].

By integrating these variables, this study provides a comprehensive analysis of the factors influencing frailty, particularly focusing on the role of social media use in maintaining or exacerbating frailty in older adults. The use of these well-established measures ensures that the study captures the multifaceted nature of frailty, encompassing both physical and psychological factors, as well as the influence of social engagement and lifestyle habits.

### 3.3. Statistical Analysis

The aim of this study was to examine the association between frailty, as measured by the Kihon Checklist, and social media use. The study first conducted an analysis of variance for numerical variables and Fisher’s exact probability tests for categorical variables to compare demographic characteristics and key variables among the three groups (frailty, pre-frailty, and non-frailty). Post hoc tests were performed only if the tests were significant. In the post hoc tests, Bonferroni correction was applied to correct for multiple comparisons in paired comparisons between groups. To achieve this, we employed ordinal logistic regression analysis, with social media usage and other frailty-related factors serving as explanatory variables influencing frailty status. Given that the dependent variable is ordinal, consisting of three categories—non-frailty, pre-frailty, and frailty—ordinal logistic regression was selected as the appropriate statistical method. This approach is suitable for modeling relationships where the outcome variable is ordered but not continuous, and it allows for an examination of how social media usage, along with other contributing factors, may predict variations in frailty across different levels of severity.

## 4. Results

In the online survey, all 333 older adults completed every question, indicating that the burden on participants was minimal. Notably, 46% of respondents selected the option “Does not apply” in response to the question, “Do you ‘like’ or ‘share’ posts on social media?” This finding suggests that at least half of the respondents use social media. Based on these results, the reliability of the survey questionnaire design was deemed sufficient, and the survey method was subsequently transitioned to a mail-based format.

### 4.1. Demographic Characteristics

**Figure 1:**
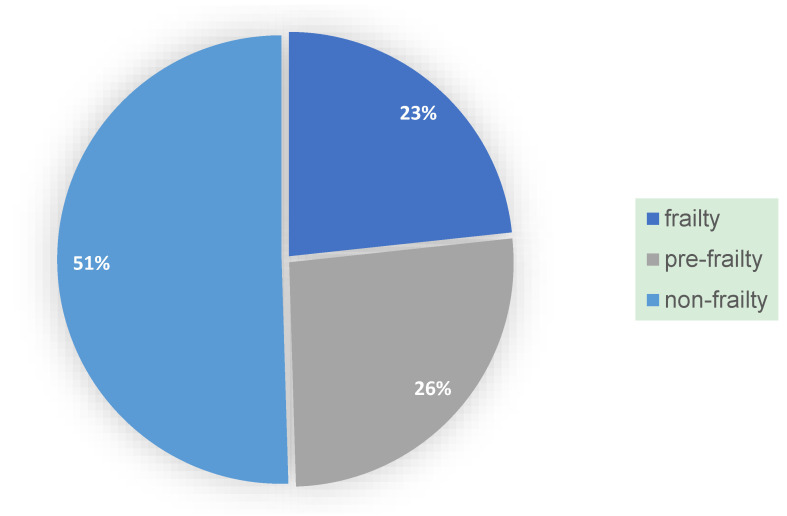
Proportions of frail, pre-frail, and non-frail individuals in the main survey.

Questionnaires were mailed to and completed by 103 individuals who consented to participate. The sample was divided into three frailty groups: frailty, pre-frailty, and non-frailty, with 12 participants in each of the frailty and pre-frailty groups, and 28 in the non-frailty group (Figure 1). The proportion of frailty individuals was 23%, and the proportion of pre-frailty individuals was 26%, totaling approximately 50%, which aligns with the overall proportion of frail and pre-frail individuals in the Japanese population as indicated [4].

As shown in Table 1, the sample included both pre-older individuals (aged 65 to 75 years) and older individuals (aged over 75 years), with an approximately equal gender distribution (52 males and 49 females). The distribution of household income and educational background also reflected the demographic profile of older adults in Japan.

Regarding household income, the most common income bracket was between JPY 3 million and JPY 4.8 million annually (37 participants). This distribution was consistent across the frailty groups, indicating no significant differences in household income by frailty status (F = 0.984, *p* = 0.592). In terms of educational background, most participants had completed vocational school or junior college (45 participants), followed by university or graduate school graduates (37 participants), with minimal representation from junior high school or high school graduates. Educational background distribution did not differ significantly across frailty groups (*p* = 0.386).

Regarding the responses to the questions on social media use and social connection, the average responses to the 1–4 scale questions are presented in Table 1. The results of Fisher’s exact test on several key variables related to social media and social participation showed significant differences between the frailty groups. Paired comparisons of post hoc analyses (Bonferroni-corrected) were also conducted to confirm differences between groups. The participants’ self-reported frequency of liking and sharing on social media use differed significantly between the groups (*p* < 0.05), with the non-frailty group reporting the highest frequency. Post-tests showed the highest frequency in the non-frail group, with significant differences identified especially between the frailty and pre-frailty groups (*p*-value < 0.01). Similarly, the ability to express feelings and thoughts on social media was significantly higher in the non-frailty group compared to the frail group (*p* < 0.01). Post hoc tests showed that the non-frail, pre-frail frail groups had significantly higher abilities when compared (*p*-value < 0.01).

Participants’ self-perceived understanding of others’ feelings and thoughts on social media also varied significantly (*p* < 0.01), with the non-frailty group again reporting higher levels of understanding. The post-test results showed a significant difference between the frailty and pre-frailty groups (adjusted *p*-value < 0.01). The self-reported friendliness to others on social media was also highest in the non-frailty group (*p* < 0.01). Post hoc tests showed that the non-frailty, pre-frailty, and frailty groups had significantly higher abilities when compared (corrected *p*-value < 0.01).

Socialization capabilities, including the ability to socialize with peers, showed significant variation across frailty groups (*p* < 0.01), with the non-frailty group again reporting greater ease in socializing. Post hoc tests showed that the non-frailty, pre-frailty and frailty groups had significantly higher abilities when compared (corrected *p*-value < 0.01). The frequency of face-to-face communication also showed a significant difference (*p* < 0.01), with those in the non-frailty group engaging in face-to-face communication more frequently than those in the frailty group. Post hoc tests showed that the non-frailty, pre-frailty, and frailty groups had significantly higher abilities when compared (corrected *p*-value < 0.01). Additionally, the frequency of engaging in favorite leisure activities was significantly higher in the non-frailty group (*p* < 0.01). Post-test results indicated a difference in the non-frailty and frailty groups (*p*-value < 0.01).

### 4.2. Ordinal Logistic Regression Analysis

The results of the ordinal logistic regression analysis, based on the Bayesian Information Criterion (BIC), highlighted key factors associated with frailty among older adults (Table 2). Personal attributes such as age, gender, household income, and educational background were controlled in the analysis. Significant associations were observed between various psychological and social engagement factors.

A strong positive correlation was found between frailty and belief in one’s ability to express thoughts and emotions on social media (β = 1.136, *p* < 0.05). These factors indicated an increased likelihood of frailty. On the contrary, factors negatively correlated with frailty included the ability to relieve stress from anxiety (β = −1.643, *p* < 0.05), active participation in non-work-related activities (β = −2.312, *p* < 0.01), engagement in community activities (β = −1.440, *p* < 0.05), satisfaction with their favorite leisure activities (β = −0.151, *p* < 0.01), satisfaction with face-to-face communication (β = −3.043, *p* < 0.05), and understanding others on social media (β = −1.791, *p* < 0.01).

The corresponding odds ratios reinforced these relationships. For instance, feelings of alienation were associated with a higher likelihood of frailty, while satisfaction with face-to-face communication and participation in non-work-related and community activities were linked to a lower risk of frailty. Additionally, the ability to understand others on social media appeared to be related to a reduced likelihood of frailty. These findings underscore the complexity of the relationship between social interactions, psychological factors, and lifestyle choices in shaping frailty risk among older adults.

## 5. Discussion

This study highlights the nuanced relationship between social media engagement and vulnerability to frailty in the older population. The way individuals interact with social media—whether through self-expression or perceived social isolation—demonstrates significant connections to their frailty risk. Importantly, lifestyle factors such as reducing stress, engaging in non-work-related outings, participating in community activities, and enjoying face-to-face interactions showed a negative correlation with frailty.

These findings align with existing literature suggesting that social media use is associated with social satisfaction and the quality of relationships [40].

The demographic attributes of the elderly participants in this study showed no significant differences when compared to the general elderly population in Japan. Similarly, the prevalence of frailty observed in this study was consistent with previous findings. Specifically, prior research reported a frailty prevalence of 8.7% and a pre-frailty prevalence of 40.8%, with minimal regional variation, particularly in the Kanto region, where frailty and pre-frailty rates were 8.0% and 39.7%, respectively [4]. In this study, the prevalence of frailty was found to be 23%, and pre-frailty was 26%. However, when combining the rates of frailty and pre-frailty, the overall prevalence of potential frailty in this study closely aligned with the results of previous studies, indicating no substantial differences.

Although no research in Japan has specifically examined the relationship between frailty and social media use, existing studies have explored the association between social media use and loneliness, a well-established factor contributing to frailty. These studies suggest that social media use is linked to reduced loneliness among older adults, which may help mitigate feelings of isolation [41]. These findings support the premise of this study, which aims to investigate the potential for interventions targeting psychological and social factors, such as loneliness, to address frailty progression in older adults. Based on these observations, the results of this study are likely to be generalizable to the broader population of older adults in Japan, particularly those residing in the Tokyo metropolitan region.

The comprehensive approach to frailty in this study highlights the significant role of communication in preventing social isolation, which is a known precursor to frailty. This study hypothesized that social media, which enables accessible online interactions, could influence the risk of frailty. The findings reveal that understanding others through social media is associated with a reduced risk of frailty, while self-expression on these platforms correlates with an increased risk. Although social media can enhance sociability [9], problematic usage patterns may lead to social isolation [42], exposing the inconsistent effects of these platforms. These results suggest that frailty is intricately linked to both social isolation and mental health challenges caused by communication deficits.

The importance of empathy in communication, particularly among older populations, also warrants attention. Research on empathy development among older university students has shown that fostering empathy can improve communication skills [43]. And previous studies have suggested that empathy, a key element of communication, has the potential to alleviate feelings of loneliness and reduce social isolation [44,45]. However, non-verbal cues—essential components of empathetic communication as outlined in Mehrabian’s principle [46]—are often absent in social media exchanges. This lack of non-verbal cues can impede empathy, complicate effective communication, and potentially reduce the frequency of in-person interactions, thereby increasing the risk of frailty. Conversely, even within the limitations of social media, the ability to understand others may still facilitate meaningful communication and help mitigate the likelihood of frailty.

Contemporary research has explored the psychosocial outcomes of social media use in older adults, indicating that social media engagement can enhance social connectedness and overall quality of life [9,40]. Yet, the impact on mental health remains multifaceted. While some evidence links high social media usage to psychological distress, it does not necessarily alleviate the effects of loneliness. Thus, addressing social isolation and fostering authentic social integration are essential for promoting mental well-being in older adults. The relationship between social media and psychosocial outcomes is complex, with studies yielding varied results. Cross-sectional research often shows positive correlations, while longitudinal studies present more ambiguous findings. Standardizing research methodologies and employing more rigorous inquiry could deepen our understanding of social media’s effects on the mental health of older populations.

In this study, a negative correlation was observed between understanding others on social media and frailty, while a positive correlation was noted between self-expression and the progression of frailty. These findings may help explain the inconsistent effects of social media on social isolation and user satisfaction reported in previous research. The impact of social media likely varies depending on how it is used. However, one limitation of this study is that it did not differentiate between specific usage methods or platforms. Future research should investigate more detailed usage patterns and platform-specific effects to clarify the diverse impacts of social media on frailty and address the inconsistencies observed in prior studies.

Another limitation of this study is the small sample size. Data collection was conducted via postal surveys, but the response rate was low, with only 103 out of 1333 participants responding (approximately 7%). The limited sample size reduced the predictive accuracy of the model equations. In future studies, it is important to address the challenges of involving older adults in research. Strategies such as reducing the number of survey items and providing clear, encouraging explanatory documents and questionnaires to facilitate participation should be implemented.

Additionally, this study did not account for certain variables that could influence the results, such as cognitive status, health status, comorbidities, and psychological changes among elderly participants. These factors may have significant effects on frailty and social media use, and their absence limits the generalizability of the findings. Future research should incorporate these variables to provide a more comprehensive understanding of the factors influencing frailty in older adults. A specific approach to address these variables would be crucial for refining interventions and improving the overall validity of this study’s conclusions.

## 6. Conclusions

This study highlights the importance of promoting both traditional social engagement and digital participation to mitigate frailty in older adults. Policies and programs designed to support older adults should focus on fostering community engagement and encouraging the responsible use of digital platforms to enhance social inclusion. Providing digital literacy training and creating accessible online environments could help older adults form and maintain social connections.

While the study demonstrates the potential benefits of social media use, further research is needed to explore the specific effects of different types of social media engagement. This includes examining how factors such as frequency, platform, and mode of interaction influence frailty and overall well-being. Longitudinal and intervention-based studies will be essential for understanding the long-term effects and establishing clearer causal relationships between social media use and frailty.

The present study provides a foundation for future research and interventions aimed at alleviating social isolation and reducing frailty, offering valuable insights into how digital and physical social participation can work together to improve the health of older adults.

## Figures and Tables

**Table 1:** Summary of demographic characteristics and variables of interest, including comparisons across frailty groups.

Variable	Total	Frailty	Pre-Frailty	Non-Frailty	*p* Value
Age	76.56	78.42	76.07	75.96	0.377
Gender					0.890
Male	52 (51%)	12 (50%)	12 (46%)	28 (55%)	
Female	49 (49%)	12 (50%)	14 (54%)	23 (45%)	
Household income	387.35 (300, 259.29)	345.95 (300, 222.85)	374.05 (300, 288.72)	415.37 (350, 263.66)	0.592
Education					0.386
Junior High School or Below	4 (4%)	1 (4%)	1 (4%)	2 (4%)	
Vocational School/Junior College Graduates	45 (44%)	13 (54%)	13 (48%)	19 (37%)	
High School Graduates	17 (17%)	2 (8%)	2 (7%)	13 (25%)	
University/Graduate School Graduates	37 (36%)	8 (33%)	11 (41%)	18 (35%)	
Do you like or share posts on social media?	2.22 (1.5, 1.33)	1.54 (1, 1.10)	2.65 (3, 1.32)	2.32 (2, 1.33)	<0.05
Do you think you can express your feelings and thoughts on social media?	2.06 (1.5, 1.19)	1.5 (1, 0.88)	2.43 (3, 1.31)	2.17 (2, 1.19)	<0.01
Do you think you understand other people’s feelings and thoughts on social media?	2.25 (3, 1.11)	1.58 (1, 0.97)	2.56 (3, 0.87)	2.43 (3, 1.17)	<0.01
Do you think you interact with others in a friendly manner on social media?	2.28 (2, 1.27)	1.58 (1, 1.18)	2.57 (3, 1.12)	2.5 (3, 1.28)	<0.01
Do you think you socialize with friends?	3.22 (3, 0.76)	2.87 (3, 0.63)	3.04 (3, 0.90)	3.47 (4, 0.64)	<0.01
Are you satisfied with face-to-face communication?	3 (3, 0.47)	2.67 (3, 0.64)	2.96 (3, 0.20)	3.18 (3, 0.39)	<0.01
Do you often engage in your favorite leisure activities?	2.99 (3, 0.92)	2.45 (3, 1.18)	2.85 (3, 0.88)	3.3 (3, 0.68)	<0.01

Notes: Data are presented as mean (median, standard deviation) or *n* (%).

**Table 2:** Results of the ordinal logistic regression analysis of this survey.

Variable	Estimate	Std. Error	Z Value	Pr [>|z|]		Odds Ratio	95% CI Lower	95% CI Upper
Relieving stress	−1.643	0.672	−2.445	0.015	*	0.193	0.047	0.674
Actively engaging in non-work-related activities	−2.312	0.918	−2.808	0.005	**	0.099	0.014	0.557
Participating in community activities	−1.440	0.513	2.207	0.027	*	0.237	0.079	0.613
Feelings of alienation	1.558	0.706	−0.216	0.829		4.750	1.253	20.57
Satisfied with favorite leisure activities	−0.151	0.699	−2.709	0.007	**	0.860	0.209	3.381
Satisfied with face-to-face communication	−3.043	1.123	−2.52	0.012	*	0.048	0.004	0.341
Belief in one’s ability to express feelings and thoughts on social media	1.136	0.528	2.149	0.031	*	3.110	1.149	9.357
Understanding others on social media	−1.791	0.677	−2.647	0.008	**	0.170	0.040	0.580

* *p* < 0.05, ** *p* < 0.01.

## Data Availability

The datasets used and/or analyzed during the current study are available from the corresponding author on reasonable request.

## Supplementary Materials

The following supporting information can be downloaded at: https://www.mdpi.com/article/10.3390/ijerph22020142/s1, Table S1: Questionnaire items.
