# Supplementary material for: A Cross-Sectional Study on the Relationship Between Social Media Use and Frailty Among the Older People in Japan
Source: Int J Environ Res Public Health. 2025 Jan 22;22(2):142. doi: 10.3390/ijerph22020142 (PMC11855460; doi:10.3390/ijerph22020142)
Supplement: Supplementary file 1 [file ijerph-22-00142-s001.zip › ijerph-3303538-supplementary.pdf]

Supplementary material Table S1

No.	Questions	Answer: Please mark one of the options with a circle	
Section 1. Kihon Checklist			
1	Do you go out alone by bus or train?	0. Yes	1. No
2	Do you do your daily shopping?	0. Yes	1. No
3	Do you manage your savings and withdrawals?	0. Yes	1. No
4	Do you visit your friends' houses?	0. Yes	1. No
5	Do you give advice to family or friends?	0. Yes	1. No
6	Can you climb stairs without holding onto a handrail or wall?	0. Yes	1. No
7	Can you stand up from a chair without holding onto anything?	0. Yes	1. No
8	Can you walk continuously for about 15 minutes?	0. Yes	1. No
9	Have you fallen in the past year?	1. Yes	0. No
10	Do you have a great fear of falling?	1. Yes	0. No
11	Have you lost 2-3 kg or more in the last six months?	1. Yes	0. No
12	Height: approx. ____ cm, Weight: approx. ____ kg. BMI = ____		
13	Has it become more difficult to eat hard foods compared to six months ago?	1. Yes	0. No
14	Do you choke on tea or soup?	1. Yes	0. No
15	Do you feel that your mouth is dry?	1. Yes	0. No
16	Do you go out at least once a week?	0. Yes	1. No
17	Have you gone out less frequently compared to last year?	1. Yes	0. No
18	Do people say you have memory problems, such as asking the same thing repeatedly?	1. Yes	0. No
19	Do you look up phone numbers and make calls on your own?	0. Yes	1. No
20	Are there times when you don't know what day it is?	1. Yes	0. No
21	(In the past two weeks) Do you feel that daily life is not fulfilling?	1. Yes	0. No
22	(In the past two weeks) Have you lost interest in things you used to enjoy?	1. Yes	0. No
23	(In the past two weeks) Do you find previously easy tasks to be burdensome?	1. Yes	0. No
24	(In the past two weeks) Do you feel that you are not a useful person?	1. Yes	0. No
25	(In the past two weeks) Do you feel tired for no reason?	1. Yes	0. No
Section 2 Frailty-Related Items			
Q1	Please provide your age.	( )	
Q2	Please indicate your gender	0. Male	1. No
Q3	Please indicate your highest level of education	1.Junior High School or below	
		2.Vocational School/Junior College Graduates	

			3.High School Graduates
			4.University/Graduate School Graduates
Q4	Please provide your approximate household income.		( )
Q5	Do you usually not eat three meals a day?	0. Yes 1. No	Sun, Y et al. 2023 [1]
Q6	Do you have fewer than 20 teeth?	0. Yes 1. No	Yamamoto T et al. 2012 [2]
Q7	Do you go for a dental check-up once every six months?	0. Yes 1. No	Kiuchi S et al. 2024 [3]
Choose from the following options:1. Strongly disagree, 2. Disagree, 3. Agree, 4. Strongly agree			
Q8	Do you think you are healthy?	1 --- 2 --- 3 --- 4	Yang, S et al 2024 [4]
Q9	Do you consciously exercise for your health?	1 --- 2 --- 3 --- 4	Yang, S et al 2024 [4]
Q10	Do you get enough rest from sleep over the past month?	1 --- 2 --- 3 --- 4	Zhang, X et al. 2020 [5]
Q11	Are you satisfied with your overall sleep?	1 --- 2 --- 3 --- 4	Zhang, X et al. 2020 [5]
Q12	Do you think you are happy?	1 --- 2 --- 3 --- 4	Ensrud, K et al 2012 [6]
Q13	Do you think you can relieve stress from worries and anxieties?	1 --- 2 --- 3 --- 4	Ensrud, K et al 2012 [6]
Q14	Do you laugh out loud often in your daily life?	1 --- 2 --- 3 --- 4	Ensrud, K et al 2012 [6]
Q15	Do you actively go out for non-work activities?	1 --- 2 --- 3 --- 4	Tsunoda, K.et al 2011 [7]
Q16	Do you participate in community activities a lot?	1 --- 2 --- 3 --- 4	Tsunoda, K.et al 2011 [7]
Q17	Do you interact with your neighbors?	1 --- 2 --- 3 --- 4	Yamashita, S et al 2021 [8]
Q18	Do you trust other people?	1 --- 2 --- 3 --- 4	Yamashita, S et al 2021 [8]
Q19	Do you think you socialize with friends?	1 --- 2 --- 3 --- 4	Maltby, J et al 2020 [9]
Q20	Do you feel alienated?	1 --- 2 --- 3 --- 4	Maltby, J et al 2020 [9]
Q21	Do you feel isolated from others?	1 --- 2 --- 3 --- 4	Maltby, J et al 2020 [9]
Q22	Do you think you engage in a variety of leisure activities?	1 --- 2 --- 3 --- 4	Yoshizawa, Y et al 2024 [10]
Q23	Do you often engage in your favorite leisure activities?	1 --- 2 --- 3 --- 4	Yoshizawa, Y et al 2024 [10].
Q24	Are you satisfied with your favorite leisure activities?	1 --- 2 --- 3 --- 4	Yoshizawa, Y et al 2024 [10].
Q25	Do you often meet and talk with people?	1 --- 2 --- 3 --- 4	Yamashita, S et al 2021 [8]
Q26	Are you satisfied with face-to-face communication?	1 --- 2 --- 3 --- 4	Yamashita, S et al 2021 [8]
Q27	Do you use social media on a daily basis?	1 --- 2 --- 3 --- 4	Yamashita, S et al 2021 [8]
Q28	Do you like or share posts on social media?	1 --- 2 --- 3 --- 4	Yamashita, S et al 2021 [8]
Q29	Do you think you can express your feelings and thoughts on social media?	1 --- 2 --- 3 --- 4	Yamashita, S et al 2021 [8]
Q30	Do you think you understand other people's feelings and thoughts on social media?	1 --- 2 --- 3 --- 4	Yamashita, S et al 2021 [8]
Q31	Do you think you interact with others in a friendly manner on social media?	1 --- 2 --- 3 --- 4	Yamashita, S et al 2021 [8]

References

1. Sun, Y.; Rong, S.; Liu, B.; Snetselaar, L.; Wallace, R.; Bao, W. Meal Skipping and Shorter Meal Intervals Are Associated with Increased Risk of All-Cause and Cardiovascular Disease Mortality among US Adults. *J. Acad. Nutr. Diet.* **2023**, *123*, 417–426. https://doi.org/10.1016/j.jand.2022.08.119.
2. Yamamoto, T.; Kondo, K.; Misawa, J.; Hirai, H.; Nakade, M.; Aida, J.; Kondo, N.; Kawachi, I.; Hirata, Y. Dental status and incident falls among older Japanese: A prospective cohort study. *BMJ Open* **2012**, *2*, e001262. https://doi.org/10.1136/bmjopen-2012-001262.
3. Kiuchi, S.; Takeuchi, K.; Saito, M.; Kusama, T.; Nakazawa, N.; Fujita, K.; Kondo, K.; Aida, J.; Osaka, K. Differences in Cumulative Long-Term Care Costs by Dental Visit Pattern Among Japanese Older Adults: The JAGES Cohort Study. *J. Gerontol. Ser. A* **2024**, *79*, glae194. https://doi.org/10.1093/gerona/glae194.
4. Yang, S.; Wu, L.; Huang, H.; Zhang, L.; Chen, Y.; Zhou, S.; Chen, X.; Wang, J.; Zhang, C.; Bao, Z. Diet and lifestyle behaviours simultaneously act on frailty: It is time to move the threshold of frailty prevention and control forward. *BMC Public Health* **2024**, *24*, 18639. https://doi.org/10.1186/s12889-024-18639-y.
5. Zhang, X.; Tan, S.S.; Franse, C.B.; Bilajac, L.; Alhambra-Borrás, T.; Garcés-Ferrer, J.; Verma, A.; Williams, G.; Clough, G.; Koppelaar, E.; et al. Longitudinal Association Between Physical Activity and Frailty Among Community-Dwelling Older Adults. *J. Am. Geriatr. Soc.* **2020**, *68*, 1484–1493. https://doi.org/10.1111/jgs.16391.
6. Ensrud, K.E.; Blackwell, T.L.; Ancoli-Israel, S.; Redline, S.; Cawthon, P.M.; Paudel, M.L.; Dam, T.-T.L.; Stone, K.L. Sleep disturbances and risk of frailty and mortality in older men. *Sleep Med.* **2012**, *13*, 1217–1225. https://doi.org/10.1016/j.sleep.2012.04.010.
7. Tsunoda, K.; Mitsuishi, Y.; Tsuji, T.; Yoon, J.; Muraki, T.; Hotta, K.; Okura, T. Association of the physical activity of community-dwelling older adults with transportation modes, depression and social networks. *Jpn. J. Geriatr.* **2011**, *48*, 516–523.
8. Yamashita, S.; Hashimoto, S.; Uno, H. A Study on Communication and Satisfaction with Leisure Activities of Older Adults—Focusing on the Use of SNS. *J. Jpn. Assoc. Incl. Soc.* **2021**, *24*, 59–69.
9. Maltby, J.; Hunt, S.A.; Ohinata, A.; Palmer, E.; Conroy, S. Frailty and Social Isolation: Comparing the Relationship between Frailty and Unidimensional and Multifactorial Models of Social Isolation. *J. Aging Health* **2020**, *32*, 1297–1308. https://doi.org/10.1177/0898264320923245.
10. Yoshizawa, Y.; Tanaka, T.; Takahashi, K.; Fuzisaki, I.; Iijima, K. The associations of frailty with regular participation in physical, cultural, and community social activities among independent elders in Japan. *N. Engl. J. Med.* **2024**, *347*, 284–287. https://doi.org/10.11236/jph.66.6_306.
